# Identification of Recombinant Human Rhinovirus A and C in Circulating Strains from Upper and Lower Respiratory Infections

**DOI:** 10.1371/journal.pone.0068081

**Published:** 2013-06-27

**Authors:** Hak Kim, Kisoon Kim, Dae-Won Kim, Hee-Dong Jung, Hyang Min Cheong, Ki Hwan Kim, Dong Soo Kim, You-Jin Kim

**Affiliations:** 1 Division of Respiratory Viruses, Center for Infectious Diseases, Korea National Institute of Health, Korea Centers for Disease Control and Prevention, Cheongwon-gun, Chungbuk, Republic of Korea; 2 Systems Biology Team, Center for Immunity and Pathology, Korea National Institute of Health, Korea Centers for Disease Control and Prevention, Cheongwon-gun, Chungbuk, Republic of Korea; 3 Department of Pediatrics, Yonsei University College of Medicine, Severance Children’s Hospital, Seodaemun-gu, Seoul, Republic of Korea; Hannover Medical School, Germany

## Abstract

Human rhinoviruses (HRVs), in the *Enterovirus* genus within the family *Picornaviridae*, are a highly prevalent cause of acute respiratory infection (ARI). Enteroviruses are genetically highly variable, and recombination between serotypes is known to be a major contribution to their diversity. Recently it was reported that recombination events in HRVs cause the diversity of HRV-C. This study analyzed parts of the viral genes spanning the 5′ non- coding region (NCR) through to the viral protein (VP) encoding sequences of 105 HRV field isolates from 51 outpatient cases of Acute Respiratory Infectious Network (ARINET) and 54 inpatient cases of severe lower respiratory infection (SLRI) surveillance, in order to identify recombination in field samples. When analyzing parts of the 5′NCR and VP4/VP2 encoding sequences, we found intra- and interspecies recombinants in field strains of HRV-A and -C. Nineteen cases of recombination events (18.1%) were found among 105 field strains. For HRV-A, there were five cases (4.8%) of intraspecies recombination events and three cases (2.8%) of interspecies recombination events. For HRV-C, there were four cases (3.8%) of intraspecies recombination events and seven cases (6.7%) of interspecies recombination events. Recombination events were significantly more frequently observed in the ARINET samples (18 cases) than in the SLRI samples (1 case; *P*< 0.0001). The recombination breakpoints were located in nucleotides (nt) 472–554, which comprise stem-loop 5 in the internal ribosomal entry site (IRES), based on the HRV-B 35 sequence (accession no. FJ445187). Our findings regarding genomic recombination in circulating HRV-A and -C strains suggest that recombination might play a role in HRV fitness and could be a possible determinant of disease severity caused by various HRV infections in patients with ARI.

## Introduction

Human rhinoviruses (HRVs), first discovered in 1953, are nonenveloped, positive single-strand RNA viruses of the genus *Enterovirus* in the family *Picornaviridae* [1,2]. HRVs are the major cause of upper and lower respiratory tract infections in humans [3]. In particular, HRVs are the second main cause of bronchiolitis and wheezing illnesses in infancy, which are strongly associated with a high risk of developing asthma in childhood [4]. It is recognized that 50–85% of most asthma exacerbations are caused by HRV infections [5,6,7,8]. HRVs are transmitted commonly by the respiratory–salivary route, both by contact and airborne transmission [9].

HRVs have a genome of approximately 7,200 base pairs (bp) containing a single reading frame that encodes four viral capsid proteins (VP1, VP2, VP3 and VP4) and seven nonstructural proteins (2A^pro^, 2B, 2C, 3A, 3B, 3C^pro^ and 3D^pol^) [8,10]. HRVs have a 5′ non-coding region (NCR) of about 650 bp that consists of a cloverleaf-like (CL) motif and an internal ribosome entry site (IRES) at the 5′ end of the genome, with roles in viral replication and translation initiation, respectively. The IRES of HRVs contains five secondary RNA structures called stem-loops (SLs) 2–6 and a polypyrimidine tract (PPT) located between SL5 and SL6 [11].

Currently, 153 proposed types of HRVs have been identified and classified into three species (A, B and C) based on the nucleotide sequences that encode the VP1 protein, as HRV-C species are difficult to isolate by *in vitro* culture and to serotype [9,12,13,14,15,16,17,18,19]. HRVs share many features of their genome organization and structure with other picornaviruses. The genus *Enterovirus* in the family *Picornaviridae* has undergone much evolutionary genetic diversification through recombination events [20,21,22,23]. HRVs have also developed genetic diversity by recombination near the 5′ NCR/P1, P1/P2 and P2/P3 boundaries [8,10,24,25]. Recombination events between the 5′ NCR and the VP4 encoding sequences have mainly been observed in HRV-C, and the recombination breakpoints have been identified at SL5 and the PPT region [10]. In addition, some HRV-C that are closely related to HRV-A based on sequence analysis of the 5′ NCR have been designated “HRV Ca”, and these HRV Ca subspecies have been suggested to arise from interspecies recombination [10,25,26]. However, the inter- or intraspecies recombination events of field HRV-A and intraspecies recombination events of field HRV-C have not been studied until recently.

After finding of HRV-C, numerous studies on epidemiological and clinical manifestations have been conducted to elucidate the pathogenicity of HRV-C infection associated with disease severity. However the correlation between disease severity and HRV-C is still controversial [27,28,29,30,31]. In addition, HRVs are genetically heterogeneous and recombination events between or within species could cause complicate the identification and typing of HRVs, as well as a differentiation of clinical consequences.

This study aimed to understand and characterize the various recombination events between 5’ NCR and VP4/VP2 region of field strains of HRV including species A, B and C, using 105 HRVs identified from two distinctive laboratory surveillance systems, the Acute Respiratory Infectious Network (ARINET) and Severe Lower Respiratory Tract Infections (SLRI) surveillances undertaken from October 2008 to March 2009. We investigated the occurrence and location of recombination events in these field strains of HRVs by phylogenetic analysis and by applying the Recombination Detection Program and described comparative analysis of unbiased recombination events in two surveillance systems with different disease severity. 

## Materials and Methods

### Ethics statement

For specimens from ARINET, this study was approved by the Institutional Review Board of Korea Centers for Disease Control and Prevention (KCDC; 2012-09CON-03-4C) as it involved de-identified remaining respiratory tract samples which were not related to human gene study and collected for the respiratory virus diagnosis with written informed consent from patients, their parents or legal guardian. De-identification was performed except for each subject’s age, gender, reported diagnosis, time of collection and virus detection results.

In the case of specimens from SLRI, ethical clearance was obtained from Yonsei University Health System Institutional Review Board, Seoul, Korea (4-2008-0649). Target population was the total population of children less than 5 years needed to be admitted for their lower respiratory infections. Patients who had their parents or legal guardians’ written consent to participate in surveillance were enrolled. We obtained their nasopharyngeal aspirate specimens and their clinical information without personal ones.

### Specimen collection and virus detection

Nasal aspirate specimens from patients with ARI (*n* = 3082) and nasopharyngeal aspirate specimens from patients with SLRI (*n* = 381) were collected in the ARINET and SLRI surveillances and marked as KA and KL in sample name respectively, in South Korea from October 2008 to March 2009 [32]. Among the HRV-positive samples-827 from ARINET and 85 from SLRI—51 and 54 samples, respectively, were selected by random sampling method. The viral RNAs of collected specimens were extracted using the QIAamp Viral RNA Mini Kits (QIAGEN, Hilden, Germany) according to the manufacturer’s instructions and stored at -70 °C until used for experiments.

The extracted RNA was applied to one-step reverse transcription–polymerase chain reaction (RT–PCR) reagents and the Labopass™ RV Detection kit (Cosmo Genetech, Seoul, South Korea) for detection of HRV. The kit was developed by the division of influenza and respiratory virus in KCDC with Cosmo Genetech [32].

### RT-PCR system for sequencing of the 5' NCR and VP4/VP2 sequences

cDNA was synthesized using SuperscriptTM [0428] Reverse Transcriptase (Invitrogen, Carlsbad, CA, USA) with oligo-dT primers according to the manufacturer[2019]s instructions. A previously reported 5[2032] NCR primer set was used to amplify part of the IRES containing SL3, SL4 and SL5 from nucleotides (nt) 176[2013]559 : based on accession number FJ445187, as shown in Table 1 [33]. From the analysis of 131 genomes of reference HRVs from Genbank and modification of previously reported primer sequences, a new primer set was designed covering the VP4/VP2 sequences (nt 447–1083).

**Table 1:** Designed Primer sets of 5′ NCR and VP4/VP2 regions.

**Primer I.D.**	**Sequence(5'->3')**	**Polarity**	**Position** ^a^
RV-OL 26	GCACTTCTGTTTCCCC	Sense	177-192
RV-OL 27	AGGACACCCAAAGTA	Antisense	544-559
RV–KH_F	CCTCCGGCCCCTGAATG**Y** ^b^GGCTAA**Y**C	Sense	447-472
RV–KH_R	GCATC**I**GG**Y**A**RY**TTCCACCACCA**N**CC**Y**TT	Antisense	1055-1083

a Position referred by Genbank accession no. FJ445187

b Bold letters mark: mix-base.

For the amplification of target genes, a 20 µl master mixture containing 2 µl of cDNA, 1 µl of each of the 10 pM target primers, 12 µl of DEPC-treated ddH_2_O, 1 µl of 2.5 mM dNTP Mix (Cosmo Genetech), 2 µl of 10X SP-Taq buffer (Cosmo Genetech) and 1 µl of SP-Taq polymerase (Cosmo Genetech) was amplified for 40 cycles of 20 s at 95 °C, 40 s at 58 °C and 1 min at 72 °C. Sequencing reactions were performed using the Big Dye Terminator Cycle Sequencing Kit and Genetic Analyzer (Applied Biosystems, Foster City, CA, USA).

### Phylogenetic analysis and recombination events prediction

The HRV sequences were aligned with those of 53 reference HRVs and previously published HRV strains: NY-074 from New York (Genbank accession number DQ875932); CL170085 from Geneva (Genbank accession number EU840952); QPM from Australia (Genbank accession number EF186077); C subtype 35 from Sweden (Genbank accession number JF436925); N10, N36 and N46 from Shanghai (Genbank accession numbers GQ223228, GQ213131 and GQ213134, respectively); LZ268, LZY79, LZ508 and LZ101 from Beijing (Genbank accession numbers JF317013, JF317014, JF317015 and JF317017, respectively); and A21_p1177_sR3307, C36_p1075_s3911 and C43_p1154_sR1124 from Wisconsin (Genbank accession numbers JN837693, JN541267 andJX074056, respectively). The Clustal W method of MegAlign (ver. 8.0.2(13.4) 2) in the Lasergene 8 program suite (DNASTAR, Madison, WI, USA) was used [34].

The 5′NCR and VP4/VP2 sequence analyses were based on nt 193–470 and nt 623–1053, respectively (Genbank accession number FJ445187) [13,25]. The phylogenetic trees of the 5′ NCR and VP4/VP2 were predicted using MEGA 4 (ver. 4.0.2) by the Neighbor-Joining method [35]. Bootstrap analysis was performed using 1000 replicates. In addition, each part of the 5′ NCR and VP4/VP2 sequences was identified using Megablast. The sequences, covering nt 193–1053, were applied to SplitsTree 4 (http://www.splitstree.org) [36] and RDP 3 (http://darwin.uvigo.es/rdp/rdp.html) for predicting recombination events [37,38].

### Nucleotide sequence accession numbers

The 19 genome sequences of recombinant viruses described in this study have been deposited in Genbank under accession numbers JX177615-JX177617, JX177619-JX177633 and JX177643.

## Results

### Clinical data and respiratory virus detection from patients

We obtained samples from the ARINET and SLRI surveillances as described in the Materials and Methods. The ARINET surveillance for outpatients with acute respiratory illness covers about 100 hospitals located all over Korea and includes all ages. The SLRI surveillance for inpatients covers four general hospitals in metropolitan areas and includes infants and children of less than 5 years of age [32]. These ARINET and SLRI surveillances represented mild and severe disease respectively, depending on clinical symptoms.

Among HRV-positive samples, 51 ARINET samples and 54 SLRI samples collected during the 2008-2009 winter season were selected for further analysis. Even though the age distributions of the ARINET and SLRI patients were not comparable directly (because the object of ARINET were patients from all ages but the SLRI from patients less than 5 years old), the majority of samples from both sets were from patients who were 1 year old or younger: 30/51 from ARINET (59%), and 43/54 from SLRI (80%), as shown in Table 2. There were no differences in the gender ratio between the ARINET (28 female and 23 male) and SLRI (28 female and 26 male) samples. Patients from the ARINET surveillance were diagnosed with pharyngitis, bronchitis, common cold, otitis media, pneumonia and sinusitis by the hospitals involved. Patients in the SLRI surveillance were diagnosed with bronchiolitis, pneumonia, croup or asthma, as shown in Table 3.

**Table 2:** The age distribution of ARINET and SLRI patients.

Age	ARINET Number (%)	SLRI Number (%)
1-12 Month	16 (31.4%)	24 (44.4%)
1 year	14 (27.5%)	19 (35.2%)
2 year	5 (9.8%)	4 (7.4%)
3-10 year	9 (17.6%)	5 (9.3%)
11-20 year	0 (0%)	−
21-50 year	5 (9.8%)	−
51-70 year	2 (3.9%)	−
Total	51	52^a^

^a^ Age of two patients in SLRI was not available

**Table 3:** The presumptive diagnosis of ARINET and SLRI patients.

	Diagnosis	Number (%)
ARINET^a^	pharyngitis	7 (12.5%)
	bronchitis	14 (25.0%)
	common cold	15 (26.8%)
	otitis media	6 (10.7%)
	pneumonia	1 (1.8%)
	sinusitis	13 (23.2%)
SLRI^b^	bronchiolitis	17 (34.0%)
	pneumonia	23 (46.0%)
	croup	6 (12.0%)
	asthma	4 (8.0%)

a Multiple diagnosis included

b Diagnosis of four patients in SLRI was not available

### Phylogenetic analysis of the VP4/VP2 sequences and 5′NCR from ARINET and SLRI

The 5′ NCR and VP4/VP2 sequences were applied to phylogenetic analysis using the MEGA 4 program with 53 reference HRVs and 14 previously isolated HRV strains, as described in the Materials and Methods. In this study, we only analyzed the sequences from 5’ NCR to VP2 region, therefore the VP4/VP2 sequences were used to define the HRV types. The phylogenetic trees predicted by the VP4/VP2 sequences were divided into HRV-A, -B and -C clusters, as shown in Figure S1A. The frequencies of presumed HRV-A, -B and -C were, respectively, 31 (60.8%), 1 (2.0%) and 19 (37.6%) in the 51 ARINET samples, and 21 (38.9%), 4 (7.4%) and 29 (53.7%) in the 54 SLRI samples. The ratios of these species differed slightly between the SLRI and ARINET samples, but the difference was not significant.

In analysis using the 5′NCR regions, the phylogenetic trees (Figure S1B) showed branches and cluster compositions differing from the VP4/VP2 sequence-based tree for both HRV-C and A. Fourteen HRV-C reference strains (QPM, QCE, NAT001, NY-074, C 24, C 25, C 26, N36, N46, C-43 p1154, CL170085, LZ269, LZY79 and LZY101), which were previously reported as Ca subspecies having a HRV-A 5′NCR, also clustered together with HRV-A reference strains. In addition, seven field strains classified as HRV-C by the VP4/VP2 tree were also clustered with HRV-A clusters in the 5′ NCR-based tree. In contrast, three field strains classed as HRV-A by the VP4/VP2 tree were classified as HRV-C in the 5′NCR-based tree. Furthermore, we found that nine field strains of HRV-A and -C that were categorized as the same species but related to a different type at the 5′NCR and VP4/VP2 sequences had possible intraspecies recombination between the same species.

**Figure 1:**
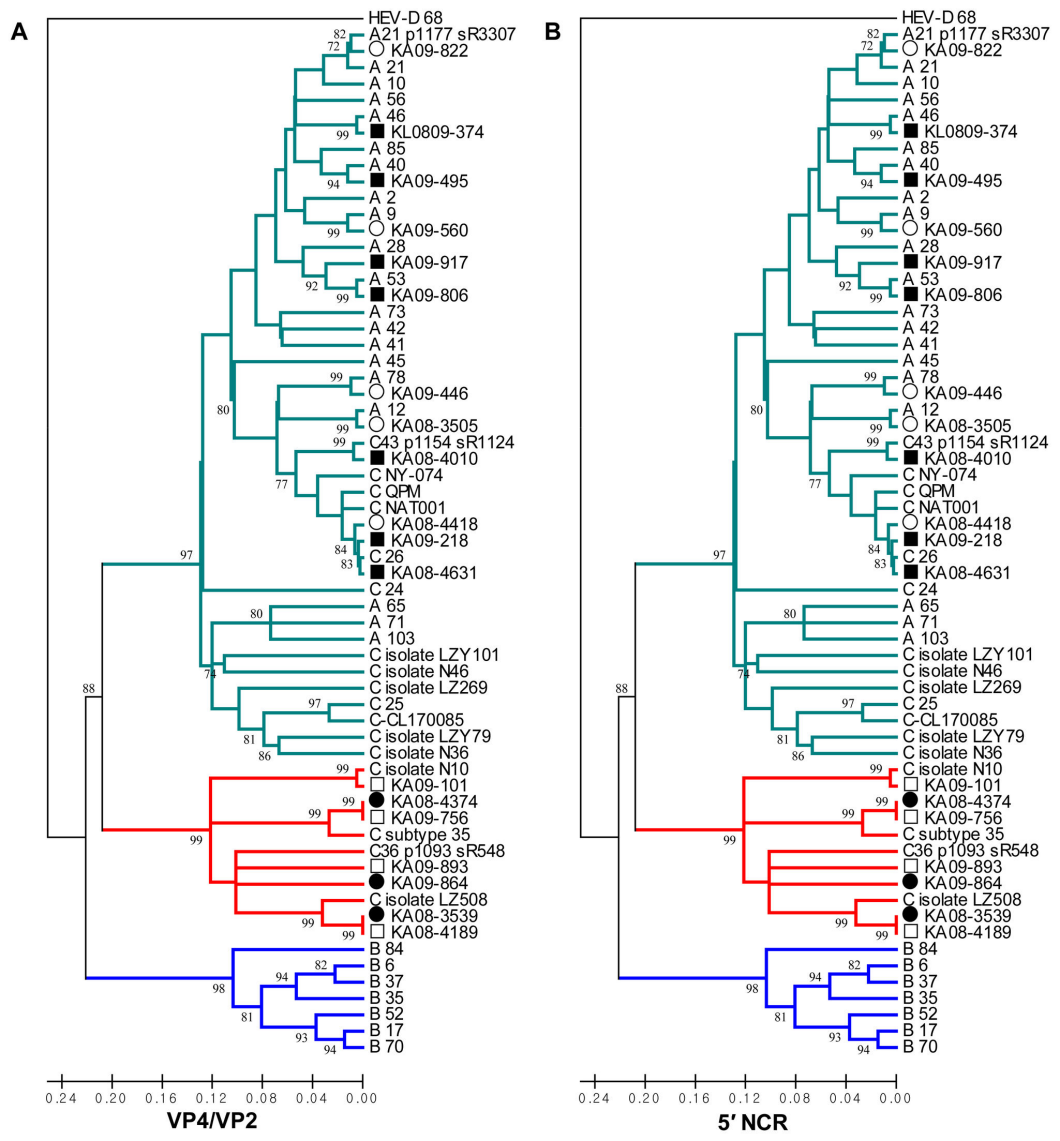
Phylogenetic analysis of field HRVs of ARINET and SLRI based on the VP4/VP2 and the 5′ NCR sequences. The phylogenetic trees were constructed using the Neighbor-Joining method in the program MEGA 4 and HEV-D 68 (accession no. AY426531) was designated as outgroup [35]. The distances of trees were computed using the Maximum Composite Likelihood method [42], and the units are the number of base substitutions per site. The intraspecies recombination strains of HRV-A, interspecies recombination strains of HRV-A, intraspecies recombination strains of HRV-C and interspecies recombination strains of HRV-C are labeled using white circles, black circles, white squares and black squares, respectively.

To further study these inconsistencies, all 5′NCR and VP4/VP2 sequences from these 19/105 (18.1%) strains were identified with the Megablast program, and showed high identities with different types in the same or different species, depending on the region analyzed. However, some reference sequences with the highest identities did not cover the entire region from the 5′NCR to the VP4/VP2 sequences. In these cases, the reference or previously published sequences having full coverage but showing slightly less identity were searched and selected as parent genomes for detecting recombination (Table 4). To summarize, only the 19 selected sequences in Table 4 were re-applied to the MEGA 4 program (Figure 1), and the phylogenetic trees showed the same results as in Figure S1. These results suggested the possibility of recombination events between the 5′ NCR and the VP4/VP2 sequences.

**Table 4:** Blast results of VP4/VP2 and 5′ NCR regions.

**Samples**	**VP4/VP2 Blast Results**	5′**NCR Blast Results**	**Classification of recombination**
	**Nearest strain** (**%** ^a^)	**Nearest strain (%)**	
KA08-3505	HRV-A 78 (92)	HRV-A 12 (98)	Intraspecies recombination of HRV-A
KA08-4418	HRV-A 56 (92)	HRV-C 26 (99)^b^	
KA09-446	HRV-A 85 (91)	HRV-A 78 (96)	
KA09-560	HRV-A 12 (94)	HRV-A 9 (97)	
KA09-822	HRV-A 45 (92)	HRV-A 21 (97)	
KA08-3539	HRV-A 43 (91)	HRV-C isolate LZ508 (95)	Interspecies recombination of HRV-A
KA08-4374	HRV-A isolate LZY172(98), HRV-A 41 (88)^c^	HRV-C isolate Resp_4817/07 (99), HRV-C subtype 35 (93)^c^	
KA09-864	HRV-A strain HRV-A21_p1177_sR3307_2010 (96)	HRV-C isolate Resp_5153/07 (99), HRV-C strain HRV-C36_p1093_sR548_2008 (83)^c^	
KA08-4189	HRV-C isolate LZY101 (99) ^b^	HRV-C isolate LZ508 (95)	Intraspecies recombination of HRV-C
KA09-101	HRV-C 26 (98)^b^	HRV-C isolate N10 (99)	
KA09-756	HRV-C isolate LZ269 (97) ^b^	HRV-C isolate Resp_4817/07 (99), HRV-C subtype 35 (93)^c^	
KA09-893	HRV-C strain CL-170085 (96) ^b^	HRV-C isolate Resp_5789/07 (97), HRV-C strain HRV-C36_p1093_sR548_2008 (84)^c^	
KA08-4010	HRV-C PHL TTa425s (98), HRV-C isolate N46 (96)^b,c^	HRV-C strain HRV-C43_p1154_sR1124_2009 (98)^b^	Interspecies recombination of HRV-C
KA08-4631	HRV-C isolate Resp_4122(98)HRV-C isolate N10 (96)^c^	HRV-C 26 (99)^b^	
KA09-218	HRV-C isolate LZY79 (99) ^b^	HRV-C 26 (99)^b^	
KA09-495	HRV-C isolate N36 (93)^b^	HRV-A 40 (98)	
KA09-806	HRV-C strain CU136(98), HRV-C sub-type 35 (82)^c^	HRV-A 53 (99)	
KA09-917	HRV-C strain NY-074 (98) ^b^	HRV-A 53 (96)	
KL0809-374	HRV-C strain Ca09-0309-U (98), HRV-C strain CL-170085 (94) ^b,c^	HRV-A 46 (98)	

The amplified sequences of VP4/VP2 and 5′NCR were identified by megablast program. 11 strains of HRV-C recombination events were identified compared to 8 strains of HRV-A recombination events. Interestingly, the recombinant viruses were mainly found in ARINET samples (94.7%: 18 of 19 strains).

a Identity

b These reference strains clustered with HRV-A in the 5′NCR phylogenetic tree in this study and previous report [10,25].

^c^ The parent genomes, that contained 5′NCR and VP4/VP2 region, of low identity for confirming recombination events

### Recombination events of HRV from field samples

Traditional bifurcating phylogenetic trees do not properly display the evolutionary history of different field strains, because one strain might be linked to more than one ancestral sequence. To confirm the possibility of inter- and intraspecies recombination, the 19 sequences were tested with the split decomposition network method and RDP3 using the regions from nt 193–1053 (from the 5′NCR to the VP2 sequence). Each sequence with a parent sequence selected from Megablast was applied to the SplitsTree 4 method [36]. All 19 selected field strains showed an interconnected relationship in the network, supporting recombination history between them, as shown in Figure S2.

**Figure 2:**
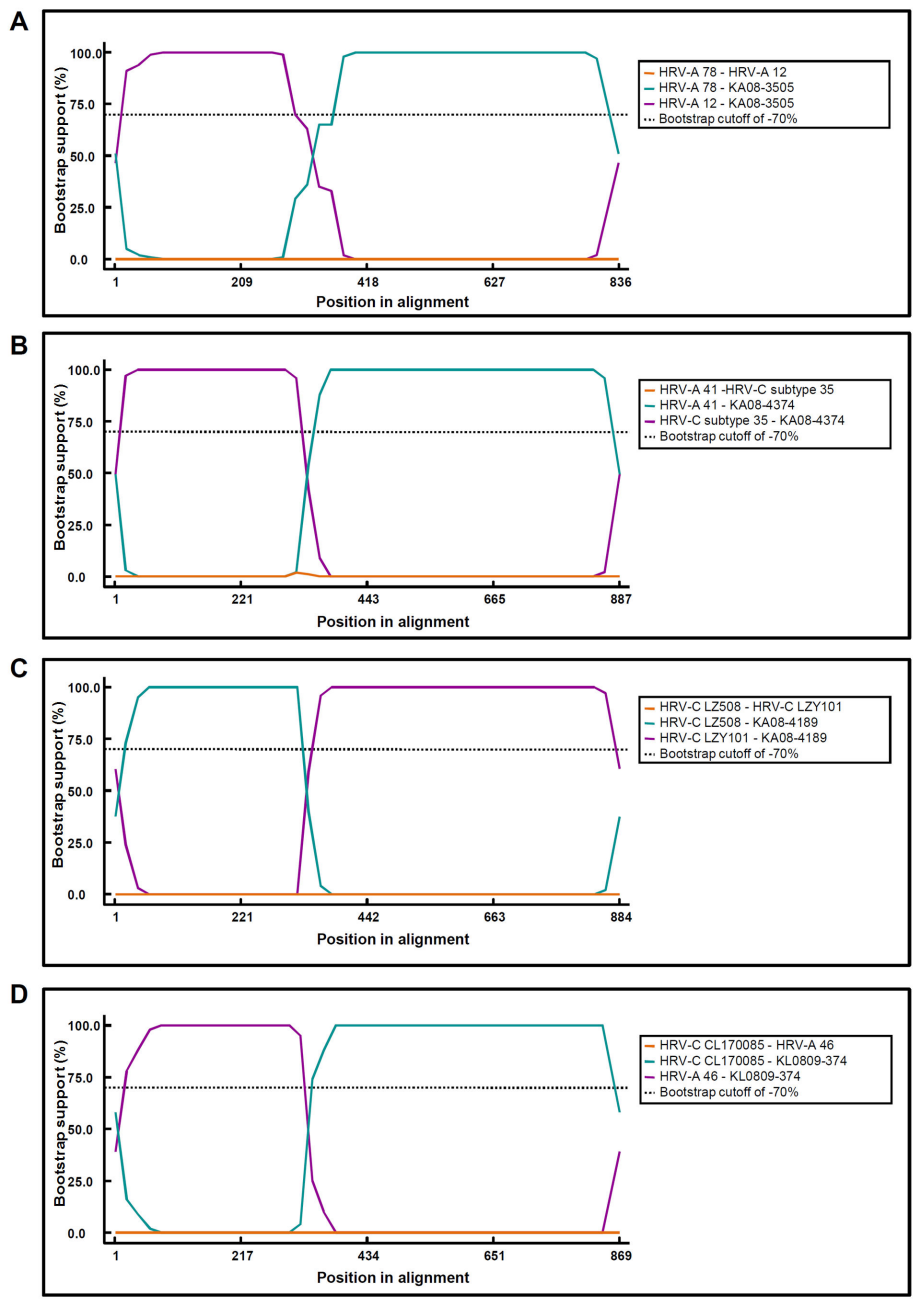
Analysis of recombination events in the 5′ NCR–VP2 sequences of HRV field isolates. The diagrams show the results of bootscan analysis in RDP. (**A**) KA08-3505 is a representative intraspecies recombinant of HRV-A which consists of the 5′ NCR sequence of HRV-A 78 and the VP sequence of HRV-A 12. (**B**) KA08-4374 is a representative interspecies recombination strain of HRV-A. The recombination event occurred between two different parent viruses: the 5′ NCR sequence of HRV-C subtype 35 and the VP sequence of HRV-A 41. (**C**) KL0809-374 was identified as an intraspecies recombination strain of HRV-C combining HRV-A 46 and HRV-C strain CL-170085. (**D**) KA08-4189 has undergone an interspecies recombination between two HRV-C viruses: the 5′ NCR sequence of HRV-C isolate LZ508 and the VP sequence of HRV-C isolate LZY101.

**Figure 3:**
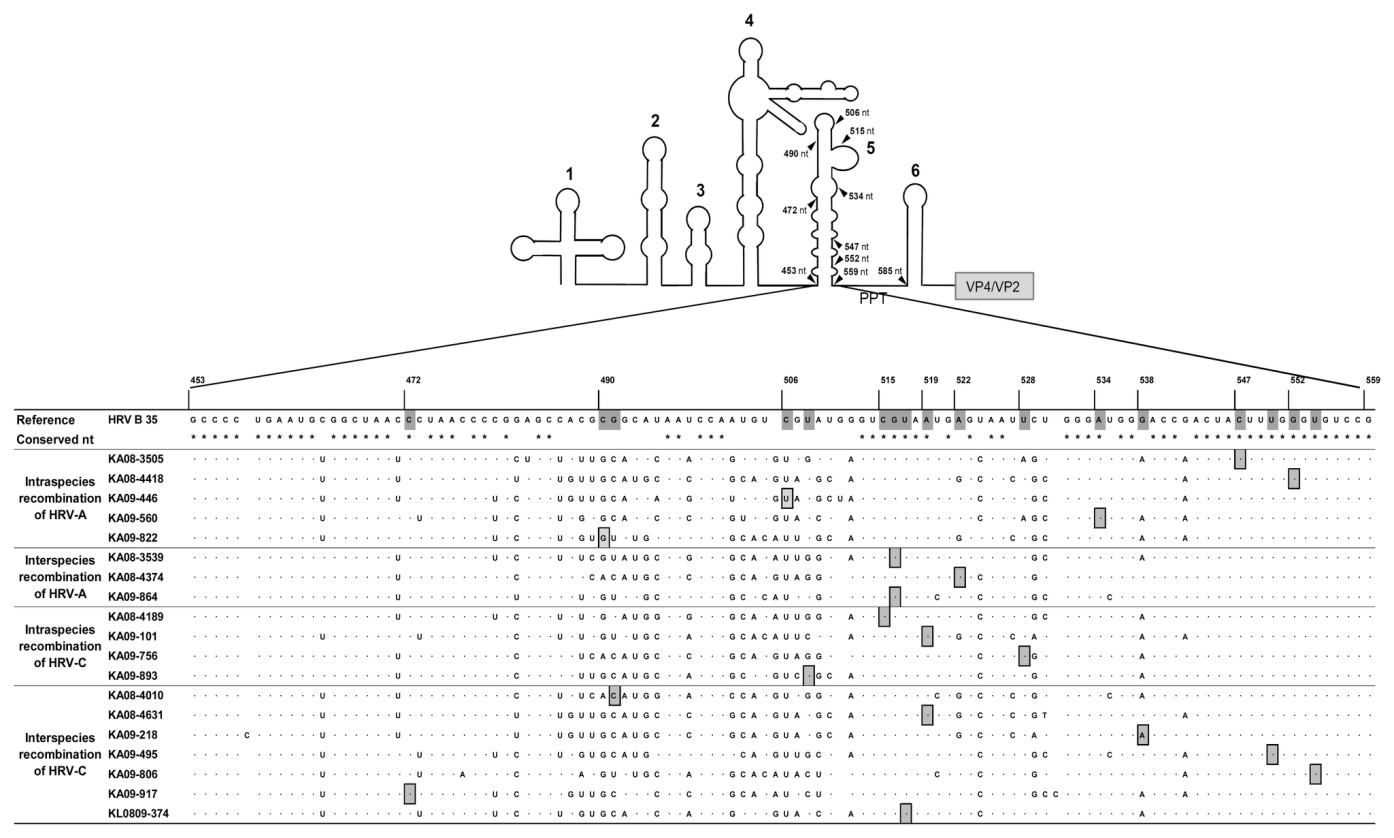
Prediction of the recombination breakpoints. Recombination breakpoints located in SL5 of the IRES (nt 472–554) were identified using RDP. The conserved nucleotides of each field strain are indicated by stars, and gray boxes show the recombination sites of each strain. The breakpoints were mainly scattered in a conserved region of SL5 (nt 515–554).

In addition, from the analysis using six methods in RDP, the 19 samples were predicted as being recombinant, and the recombination breakpoints were also suggested as expected from the results of phylogenetic analysis (Figure 2 and Table 5). The breakpoint indicated by at least three methods was identified as the breakpoint of each recombination strain, as shown in Figure 3. Strains KA08-3505, KA08-4418, KA09-446, KA09-560 and KA09-822 had an intraspecies recombination of HRV-A at nt positions 547, 552, 506, 534 and 490, respectively. In interspecies recombination of HRV-A strains, the recombination breakpoints of the KA08-3539, KA09-4374 and KA09-864 strains were identified at nt positions 516, 522 and 516, respectively. The nt positions 591, 519, 538, 550, 554, 472 and 517 were identified as the recombination breakpoints of KA08-4010, KA08-4631, KA09-218, KA09-495, KA09-806, KA09-917 and KL0809-374, respectively, in interspecies recombination strains of the HRV-C virus. KA08-4189, KA09-101, KA09-756 and KA09-893 have breakpoints at nt positions 515, 519, 528 and 452, respectively, in intraspecies recombination strains of the HRV-C virus. Most of the breakpoints were scattered at the conserved region of SL5 from nt 515–554. SL5, along with the PPT, is known as the binding region for the cellular translation initiation protein: polypyrimidine tract binding protein (PTB).

**Table 5:** Analysis results of recombination break points in recombinant viruses.

**Samples**	**RDP**		**Genconv**		**Bootscan**		**MaxChi**		**Chimaera**		**3Seq**		**Classification of recombination**
	**break point**	***P*-value**	**break point**	***P*-value**	**break point**	***P*-value**	**break point**	***P*-value**	**break point**	***P*-value**	**break point**	***P*-value**	
KA08-3505	547^a^	1.746X10^-12^	547	3.405X10^-13^	664	4.481X10^-14^	547	6.250X10^-11^	547	1.483X10^-12^	547	2.220X10^-16^	Intraspecies recombination of HRV-A
KA08-4418	552	1.016X10^-28^	552	3.904X10^-27^	552	3.398X10^-29^	552	5.081X10^-18^	552	1.334X10^-17^	552	9.208X10^-35^	
KA09-446	506	6.793X10^-13^	508	4.366X10^-11^	606	2.668X10^-13^	506	4.664X10^-12^	506	1.724X10^-12^	506	1.224X10^-14^	
KA09-560	534	7.549X10^-15^	519	1.330X10^-13^	534	2.177X10^-14^	534	4.617X10^-14^	534	2.416X10^-15^	519	1.994X10^-23^	
KA09-822	490	5.139X10^-18^	490	2.442X10^-17^	498	8.780X10^-18^	490	2.442X10^-14^	490	2.333X10^-13^	490	5.371X10^-24^	
KA08-3539	566	1.701X10^-23^	516	1.216X10^-22^	516	2.547X10^-24^	566	4.391X10^-20^	516	1.880X10^-21^	516	5.423X10^-39^	Interspecies recombination of HRV-A
KA08-4374	522	5.315X10^-26^	522	1.317X10^-22^	522	7.161X10^-23^	534	2.339X10^-19^	526	1.461X10^-21^	526	1.923X10^-46^	
KA09-864	507	1.614X10^-20^	516	2.666X10^-20^	516	3.225X10^-21^	516	2.470X10^-15^	516	1.568X10^-18^	507	1.641X10^-32^	
KA08-4189	515	1.212X10^-25^	515	6.197X10^-25^	515	1.574X10^-20^	549	8.018X10^-21^	515	1.039X10^-22^	515	1.451X10^-46^	Intraspecies recombination of HRV-C
KA09-101	undetermind	undetermind	519	1.010X10^-26^	519	3.573X10^-26^	519	2.994X10^-22^	519	4.491X10^-22^	519	2.313X10^-60^	
KA09-756	528	3.465X10^-22^	528	1.170X10^-21^	506	9.908X10^-19^	550	7.378X10^-21^	550	4.684X10^-22^	528	1.995X10^-39^	
KA09-893	452	1.243X10^-16^	508	1.896X10^-17^	508	7.852X10^-19^	508	8.768X10^-13^	508	7.926X10^-15^	508	2.160X10^-18^	
KA08-4010	491	2.038X10^-21^	484	3.907X10^-22^	491	6.837X10^-22^	491	2.019X10^-17^	491	5.843X10^-18^	484	4.683X10^-32^	Interspecies recombination of HRV-C
KA08-4631	519	2.001X10^-31^	519	1.995X10^-28^	519	1.546X10^-33^	519	5.949X10^-23^	519	1.111X10^-22^	519	7.058X10^-50^	
KA09-218	undetermind	undetermind	538	1.590X10^-30^	538	1.318X10^-34^	538	1.032X10^-23^	538	5.459X10^-24^	538	1.572X10^-59^	
KA09-495	550	9.932X10^-27^	550	3.055X10^-27^	563	1.810x10^-26^	550	2.229X10^-18^	550	2. 837X10^-20^	553	4.557X10^-40^	
KA09-806	554	2.175X10^-26^	554	9.415X10^-31^	554	5.104X10^-33^	554	1.246X10^-22^	588	1.314X10^-21^	554	7.437X10^-32^	
KA09-917	undetermind	undetermind	472	3.654X10^-15^	472	6.307X10^-18^	515	3.314X10^-14^	515	9.148X10^-14^	472	1.178X10^-20^	
KL0809-374	517	7.58510^-16^	517	9.589X10^-13^	517	8.460X10^-15^	517	1.822X10^-17^	517	4.989X10^-19^	517	2.532X10^-42^	

Results are shown for all recombination events with 6 analysis methods by RDP program. ^a^ : Position referred by GenBank accession no. FJ445187

To summarize, 105 field strains were characterized. Among these, five cases (4.8%) of intraspecies recombination strains and three cases (2.8%) of interspecies recombination strains were found among HRV-A viruses, and four cases (3.8%) of intraspecies recombination strains and seven cases (6.7%) of interspecies recombination strains were identified among HRV-C viruses. Recombination events were significantly more frequent in the ARINET samples (18/54; 33%) than in the SLRI samples (1/51; 2%; *P*< 0.0001).

## Discussion

In our study, 52 strains of HRV-A (49.5%) and 48 strains of HRV-C (45.7%) were identified in 105 field samples, whereas only five HRV-B viruses (4.8%) were found. There was no clustering difference in the distribution of ARINET and SLRI strains representing acute, mild and severe illness. Coinfection with another respiratory virus—mainly RSV—was found in 10 cases of HRV-A, 15 cases of HRV-C and four cases of HRV-B.

HRVs have remarkable genetic and antigenic variability, 102 known serotypes of HRV-A and B, and new types of HRV-C are being discovered continually. It is known that recombination events in the HRV genome have increased the diversity of each virus in the family *Picornaviridae* [20,21,22,23]. In earlier studies, Lee et al. (2007) analyzed the 5′NCR of 103 HRVs from Wisconsin and confirmed nine novel field strains [13,25]. In 2009, Huang et al. referred to these results and studied recombinant HRV-C among 66 HRVs from cases of ARI. In that study, 14 of 34 strains of HRV-C were found to be related closely to HRV-A by analysis of the 5′ NCR sequences, and these strains of HRV-C were designated as “HRV Ca”. The authors suggested that this strain had arisen from interspecies recombination, and that the Cc subspecies containing the HRV-C 5′NCR and VP4/VP2 sequences had not experienced a recombination event [10,25]. Palmenberg et al. identified intraspecies recombination of the HRV-A strain in three HRV-A reference viruses and interspecies recombination events in HRV-C viruses by phylogenetic and recombinant detection analysis [8,10,24,25]. In the present study, intraspecies recombination events of HRV-A and interspecies recombination events of HRV-C were also detected using similar methods. Surprisingly, we also detected three cases (2.8%) of interspecies recombination in HRV-A and four cases (3.8%) of intraspecies recombination in HRV-C viruses. The incidence of recombination events was found to be similarly distributed in both HRV-C (11/48 cases) and HRV-A (8/52 cases) strains (*P*>0.05) by testing for any difference between the two proportions [39]. We also confirmed that interspecies recombination events had occurred in 14 reference strains of HRV-C reference strains: QPM, QCE, NAT001, NY-074, C 24, C 25, C 26, N36, N46, C-43 p1154, CL170085, LZ269, LZY79 and LZY101. Interestingly, KA08-3539 and KA08-4189 have the same nucleotides in the 5′NCR regions which clustered with the C isolate LZ508, but have the different VP4/VP2 sequences. Thus, KA08-3539 (interspecies recombination in HRV-A) and KA08-4189 (intraspecies recombination in HRV-C) were related to HRV-A 42 and to the C isolate LZY101, respectively. KA08-4374 (interspecies recombination in HRV-A) and KA09-756 (intraspecies recombination in HRV-C) also had the same 5′ NCR and differences in the VP4/VP2 sequences. We assume that recombination events in the 5′NCR between VP4 and VP2 could have led to this diversity of HRVs.

The recombinant breakpoint was located at the IRES in SL5 at nt 472–554, as shown in Table 5 and Figure 3. McIntyre et al. have identified the recombination breakpoints, which were at SL5 (nt 484–548, based on accession number FJ445187) and PPT–SL6 (nt 560–581), of an interspecies recombinant rhinovirus of HRV-C [10]. In our study, although the breakpoint was not identified at PPT, the breakpoints in SL5 were similar to McIntyre’s results. In addition, most of the breakpoints were scattered at nt 515–554 (the conserved nucleotide region in SL5), as shown in Figure 3. The 5′NCR including the IRES plays an important role during viral replication, transcription and translation through the construction of a secondary RNA structure. The SL5 region of the 5′ NCR forms an RNA–protein complex with PTB, the cellular translation initiation protein, and it is known that the efficiency of PTB in stimulating IRES activity is affected by variations in IRES structure in polioviruses [40].

Recently, artificial 5′ NCR interspecies strains produced by recombination between enteroviruses and rhinoviruses were investigated for studying the efficiency of the 5′ NCR in translation and replication *in vitro*. This study showed that the genome of the field virus was more efficient with translation and replication than the artificial recombination genomes [41]. Accordingly, we assume that the translation and replication efficiencies are generally decreased by recombination events during evolution, except for a few favorable combinations and the recombinant viruses may have a different optimal temperature resulting to upper and lower respiratory tract infections. Although genetic and immunological predispositions of patients are primary contributor for determination of disease severity, current results presented here also support that hypothesis, with 18 cases of recombinant viruses in the ARINET isolates but only one case in the SLRI isolates. Another suggestion from the current hypothesis is that sequence information of 5’ NCR and recombination characteristics may lead us to identify a closer relationship between viral diversity and disease severity rather than single criteria of HRVs classification based on VP region sequences.

In conclusion, this study is the first report describing intra- and interspecies genomic recombination in circulating HRV-A and -C isolated from patients with acute or severe respiratory illness and these results will assist in investigating the causes of the diversity and evolution of HRVs arising through recombination events. Further study should be required on the correlation between recombination at SL5 and the assignment of virulence factor(s) in recombinant viruses to elucidate the public health impact of HRV diversity.

## Supporting Information

Figure S1 Phylogenetic tree with 53 reference HRVs and 14 previously isolated HRV strains NY-074 from New york (USA), CL170085 from Geneva (Switzerland), QPM from Australia, C subtype 35 from Sweden, N10, N36, and N46 from Shanghai, China, LZ268, LZY79, LZ508, and LZ101 from Beijing, China and A21_p1177_sR3307, C36_p1075_s3911, and C43_p1154_sR1124 from Wisconsin (USA) based on VP4/VP2 regions (**A**) and 5′ NCR regions (**B**) of human rhinovirus. The trees were constructed by 'Neighbor-Joining' method in the MEGA 4 program and HEV-D 68 strain was used as outgroup. The distances of trees were computed using the Maximum Composite Likehood method and were the units of the number of base substitutions per site. The intraspecies recombination strains of HRV-A, interspecies recombination strains of HRV-A, intraspecies recombination strains of HRV-C and interspecies recombination strains of HRV-C are labeled using white circles, black circles, white squares and black squares, respectively.
(DOCX)

Figure S2 The splits trees were predicted by decomposition network method in SplitsTree 4 (http://www.splitstree.org). 19 recombination strains showed the interconnected relationship in the network and supporting recombination between them. (**A**) The intraspecies recombinant strains of HRV-A, (**B**) interspecies recombinant strains of HRV-A, (**C**) intraspecies recombinant strains of HRV-C, (**D**) interspecies recombinant strains of HRV-C.
(DOCX)

## References

[1] Price WH (1956) The Isolation of a New Virus Associated with Respiratory Clinical Disease in Humans. Proc Natl Acad Sci U S A 42: 892-896. doi:10.1073/pnas.42.12.892. PubMed: 16589969. 16589969 10.1073/pnas.42.12.892 PMC528365

[2] Pelon W, Mogabgab WJ, Phillips IA, Pierce WE (1957) A cytopathogenic agent isolated from naval recruits with mild respiratory illnesses. Proc Soc Exp Biol Med 94: 262-267. PubMed: 13408229. 13408229 10.3181/00379727-94-22915

[3] Mackay IM (2008) Human rhinoviruses: the cold wars resume. J Clin Virol 42: 297-320. doi:10.1016/j.jcv.2008.04.002. PubMed: 18502684. 18502684 10.1016/j.jcv.2008.04.002 PMC7108405

[4] Jackson DJ, Gangnon RE, Evans MD, Roberg KA, Anderson EL et al. (2008) Wheezing rhinovirus illnesses in early life predict asthma development in high-risk children. Am J Respir Crit Care Med 178: 667-672. doi:10.1164/rccm.200802-309OC. PubMed: 18565953. 18565953 10.1164/rccm.200802-309OC PMC2556448

[5] Gern JE, Busse WW (1999) Association of rhinovirus infections with asthma. Clin Microbiol Rev 12: 9-18. PubMed: 9880472. 9880472 10.1128/cmr.12.1.9 PMC88904

[6] Hayden FG (2004) Rhinovirus and the lower respiratory tract. Rev Med Virol 14: 17-31. doi:10.1002/rmv.406. PubMed: 14716689. 14716689 10.1002/rmv.406 PMC7169234

[7] Dougherty RH, Fahy JV (2009) Acute exacerbations of asthma: epidemiology, biology and the exacerbation-prone phenotype. Clin Exp Allergy 39: 193-202. doi:10.1111/j.1365-2222.2008.03157.x. PubMed: 19187331. 19187331 10.1111/j.1365-2222.2008.03157.x PMC2730743

[8] Palmenberg AC, Rathe JA, Liggett SB (2010) Analysis of the complete genome sequences of human rhinovirus. J Allergy Clin Immunol 125: 1190-1199; quiz 1200-1191. doi:10.1016/j.jaci.2010.04.010. PubMed : 20471068 20471068 10.1016/j.jaci.2010.04.010 PMC2893015

[9] Wisdom A, Leitch EC, Gaunt E, Harvala H, Simmonds P (2009) Screening respiratory samples for detection of human rhinoviruses (HRVs) and enteroviruses: comprehensive VP4-VP2 typing reveals high incidence and genetic diversity of HRV species C. J Clin Microbiol 47: 3958-3967. doi:10.1128/JCM.00993-09. PubMed: 19828751. 19828751 10.1128/JCM.00993-09 PMC2786677

[10] McIntyre CL, McWilliam Leitch EC, Savolainen-Kopra C, Hovi T, Simmonds P (2010) Analysis of genetic diversity and sites of recombination in human rhinovirus species C. J Virol 84: 10297-10310. doi:10.1128/JVI.00962-10. PubMed: 20668080. 20668080 10.1128/JVI.00962-10 PMC2937769

[11] Borman A, Jackson RJ (1992) Initiation of translation of human rhinovirus RNA: mapping the internal ribosome entry site. Virology 188: 685-696. doi:10.1016/0042-6822(92)90523-R. PubMed: 1316679. 1316679 10.1016/0042-6822(92)90523-r

[12] Hughes PJ, North C, Jellis CH, Minor PD, Stanway G (1988) The nucleotide sequence of human rhinovirus 1B: molecular relationships within the rhinovirus genus. J Gen Virol 69(1): 49-58. doi:10.1099/0022-1317-69-1-49. 2826669 10.1099/0022-1317-69-1-49

[13] Lee WM, Kiesner C, Pappas T, Lee I, Grindle K et al. (2007) A diverse group of previously unrecognized human rhinoviruses are common causes of respiratory illnesses in infants. PLOS ONE 2: e966. doi:10.1371/journal.pone.0000966. PubMed: 17912345. 17912345 10.1371/journal.pone.0000966 PMC1989136

[14] Savolainen C, Blomqvist S, Mulders MN, Hovi T (2002) Genetic clustering of all 102 human rhinovirus prototype strains: serotype 87 is close to human enterovirus 70. J Gen Virol 83: 333-340. PubMed: 11807226. 11807226 10.1099/0022-1317-83-2-333

[15] Arden KE, McErlean P, Nissen MD, Sloots TP, Mackay IM (2006) Frequent detection of human rhinoviruses, paramyxoviruses, coronaviruses, and bocavirus during acute respiratory tract infections. J Med Virol 78: 1232-1240. doi:10.1002/jmv.20689. PubMed: 16847968. 16847968 10.1002/jmv.20689 PMC7167201

[16] Lamson D, Renwick N, Kapoor V, Liu Z, Palacios G et al. (2006) MassTag polymerase-chain-reaction detection of respiratory pathogens, including a new rhinovirus genotype, that caused influenza-like illness in New York State during 2004-2005. J Infect Dis 194: 1398-1402. doi:10.1086/508551. PubMed: 17054069. 17054069 10.1086/508551 PMC7110122

[17] Lau SK, Yip CC, Tsoi HW, Lee RA, So LY et al. (2007) Clinical features and complete genome characterization of a distinct human rhinovirus (HRV) genetic cluster, probably representing a previously undetected HRV species, HRV-C, associated with acute respiratory illness in children. J Clin Microbiol 45: 3655-3664. doi:10.1128/JCM.01254-07. PubMed: 17804649. 17804649 10.1128/JCM.01254-07 PMC2168475

[18] Simmonds P, McIntyre C, Savolainen-Kopra C, Tapparel C, Mackay IM et al. (2010) Proposals for the classification of human rhinovirus species C into genotypically assigned types. J Gen Virol 91: 2409-2419. doi:10.1099/vir.0.023994-0. PubMed: 20610666. 20610666 10.1099/vir.0.023994-0

[19] McIntyre CL, Savolainen-Kopra C, Hovi T, Simmonds P (2013) Recombination in the evolution of human rhinovirus genomes. Arch Virol. PubMed: 23443931. 10.1007/s00705-013-1634-6 23443931

[20] Mulders MN, Salminen M, Kalkkinen N, Hovi T (2000) Molecular epidemiology of coxsackievirus B4 and disclosure of the correct VP1/2A(pro) cleavage site: evidence for high genomic diversity and long-term endemicity of distinct genotypes. J Gen Virol 81: 803-812. PubMed: 10675418. 10675418 10.1099/0022-1317-81-3-803

[21] Smura T, Blomqvist S, Paananen A, Vuorinen T, Sobotová Z et al. (2007) Enterovirus surveillance reveals proposed new serotypes and provides new insight into enterovirus 5'-untranslated region evolution. J Gen Virol 88: 2520-2526. doi:10.1099/vir.0.82866-0. PubMed: 17698662. 17698662 10.1099/vir.0.82866-0

[22] Yozwiak NL, Skewes-Cox P, Gordon A, Saborio S, Kuan G et al. (2010) Human enterovirus 109: a novel interspecies recombinant enterovirus isolated from a case of acute pediatric respiratory illness in Nicaragua. J Virol 84: 9047-9058. doi:10.1128/JVI.00698-10. PubMed: 20592079. 20592079 10.1128/JVI.00698-10 PMC2937614

[23] Lukashev AN (2005) Role of recombination in evolution of enteroviruses. Rev Med Virol 15: 157-167. doi:10.1002/rmv.457. PubMed: 15578739. 15578739 10.1002/rmv.457

[24] Palmenberg AC, Spiro D, Kuzmickas R, Wang S, Djikeng A et al. (2009) Sequencing and analyses of all known human rhinovirus genomes reveal structure and evolution. Science 324: 55-59. doi:10.1126/science.1165557. PubMed: 19213880. 19213880 10.1126/science.1165557 PMC3923423

[25] Huang T, Wang W, Bessaud M, Ren P, Sheng J et al. (2009) Evidence of recombination and genetic diversity in human rhinoviruses in children with acute respiratory infection. PLOS ONE 4: e6355. doi:10.1371/journal.pone.0006355. PubMed: 19633719. 19633719 10.1371/journal.pone.0006355 PMC2712091

[26] Bochkov YA, Gern JE (2012) Clinical and molecular features of human rhinovirus C. Microbes Infect 14: 485-494. doi:10.1016/j.micinf.2011.12.011. PubMed: 22285901. 22285901 10.1016/j.micinf.2011.12.011 PMC3351075

[27] Piralla A, Rovida F, Campanini G, Rognoni V, Marchi A et al. (2009) Clinical severity and molecular typing of human rhinovirus C strains during a fall outbreak affecting hospitalized patients. J Clin Virol 45: 311-317. doi:10.1016/j.jcv.2009.04.016. PubMed: 19473873. 19473873 10.1016/j.jcv.2009.04.016

[28] Renwick N, Schweiger B, Kapoor V, Liu Z, Villari J et al. (2007) A recently identified rhinovirus genotype is associated with severe respiratory-tract infection in children in Germany. J Infect Dis 196: 1754-1760. doi:10.1086/524312. PubMed: 18190255. 18190255 10.1086/524312 PMC7109967

[29] Smuts HE, Workman LJ, Zar HJ (2011) Human rhinovirus infection in young African children with acute wheezing. BMC Infect Dis 11: 65. doi:10.1186/1471-2334-11-65. PubMed: 21401965. 21401965 10.1186/1471-2334-11-65 PMC3065410

[30] Wisdom A, Kutkowska AE, McWilliam Leitch EC, Gaunt E, Templeton K et al. (2009) Genetics, recombination and clinical features of human rhinovirus species C (HRV-C) infections; interactions of HRV-C with other respiratory viruses. PLOS ONE 4: e8518. doi:10.1371/journal.pone.0008518. PubMed: 20041158. 20041158 10.1371/journal.pone.0008518 PMC2794544

[31] Landa-Cardeña A, Morales-Romero J, García-Roman R, Cobián-Güemes AG, Méndez E et al. (2012) Clinical characteristics and genetic variability of human rhinovirus in Mexico. Viruses 4: 200-210. doi:10.3390/v4020200. PubMed: 22470832. 22470832 10.3390/v4020200 PMC3315212

[32] Chun JK, Lee JH, Kim HS, Cheong HM, Kim KS et al. (2009) Establishing a surveillance network for severe lower respiratory tract infections in Korean infants and young children. Eur J Clin Microbiol Infect Dis 28: 841-844. doi:10.1007/s10096-009-0701-0. PubMed: 19190941. 19190941 10.1007/s10096-009-0701-0 PMC7088216

[33] Papadopoulos NG, Sanderson G, Hunter J, Johnston SL (1999) Rhinoviruses replicate effectively at lower airway temperatures. J Med Virol 58: 100-104. doi:10.1002/(SICI)1096-9071(199905)58:1. PubMed: 10223554. 10223554 10.1002/(sici)1096-9071(199905)58:1<100::aid-jmv16>3.0.co;2-d

[34] Thompson JD, Higgins DG, Gibson TJ (1994) CLUSTAL W: improving the sensitivity of progressive multiple sequence alignment through sequence weighting, position-specific gap penalties and weight matrix choice. Nucleic Acids Res 22: 4673-4680. doi:10.1093/nar/22.22.4673. PubMed: 7984417. 7984417 10.1093/nar/22.22.4673 PMC308517

[35] Saitou N, Nei M (1987) The neighbor-joining method: a new method for reconstructing phylogenetic trees. Mol Biol Evol 4: 406-425. PubMed: 3447015. 3447015 10.1093/oxfordjournals.molbev.a040454

[36] Huson DH, Bryant D (2006) Application of phylogenetic networks in evolutionary studies. Mol Biol Evol 23: 254-267. PubMed: 16221896. 16221896 10.1093/molbev/msj030

[37] McVean GA, Myers SR, Hunt S, Deloukas P, Bentley DR et al. (2004) The fine-scale structure of recombination rate variation in the human genome. Science 304: 581-584. doi:10.1126/science.1092500. PubMed: 15105499. 15105499 10.1126/science.1092500

[38] Martin DP, Lemey P, Lott M, Moulton V, Posada D et al. (2010) RDP3: a flexible and fast computer program for analyzing recombination. Bioinformatics 26: 2462-2463. doi:10.1093/bioinformatics/btq467. PubMed: 20798170. 20798170 10.1093/bioinformatics/btq467 PMC2944210

[39] Liu GF (2012) A note on effective sample size for constructing confidence intervals for the difference of two proportions. Pharm Stat 11: 163-169. doi:10.1002/pst.540. PubMed: 22337507. 22337507 10.1002/pst.540

[40] Kafasla P, Morgner N, Robinson CV, Jackson RJ (2010) Polypyrimidine tract-binding protein stimulates the poliovirus IRES by modulating eIF4G binding. EMBO J 29: 3710-3722. doi:10.1038/emboj.2010.231. PubMed: 20859255. 20859255 10.1038/emboj.2010.231 PMC2982756

[41] Schibler M, Gerlach D, Martinez Y, Belle SV, Turin L et al. (2012) Experimental human rhinovirus and enterovirus interspecies recombination. J Gen Virol 93: 93-101. doi:10.1099/vir.0.035808-0. PubMed: 21940413. 21940413 10.1099/vir.0.035808-0

[42] Tamura K, Nei M, Kumar S (2004) Prospects for inferring very large phylogenies by using the neighbor-joining method. Proc Natl Acad Sci U S A 101: 11030-11035. doi:10.1073/pnas.0404206101. PubMed: 15258291. 15258291 10.1073/pnas.0404206101 PMC491989

