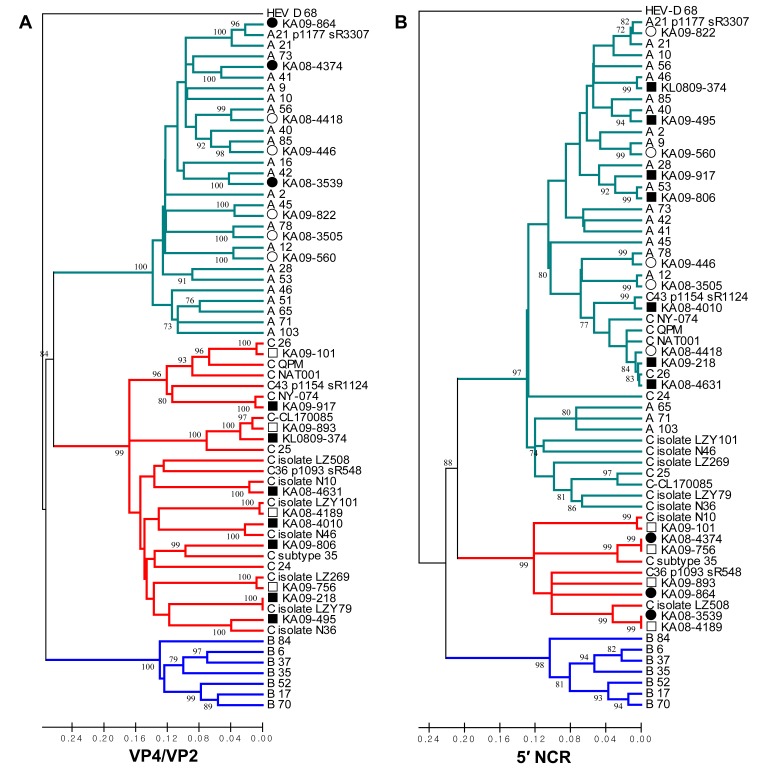# Correction: Identification of Recombinant Human Rhinovirus A and C in Circulating Strains from Upper and Lower Respiratory Infections

**DOI:** 10.1371/annotation/76668fbc-62a5-4175-939e-d5cfae5c2f59

**Published:** 2014-01-13

**Authors:** Hak Kim, Kisoon Kim, Dae-Won Kim, Hee-Dong Jung, Hyang Min Cheong, Ki Hwan Kim, Dong Soo Kim, You-Jin Kim

Figure 1A is incorrect. Please see the corrected Figure 1 here: 

**Figure:**