# Supplementary material for: Identification of Recombinant Human Rhinovirus A and C in Circulating Strains from Upper and Lower Respiratory Infections
Source: PLoS One. 2013 Jun 27;8(6):e68081. doi: 10.1371/journal.pone.0068081 (PMC3695095; doi:10.1371/journal.pone.0068081)
Supplement: Figure S1 — Phylogenetic tree with 53 reference HRVs and 14 previously isolated HRV strains NY-074 from New york (USA), CL170085 from Geneva (Switzerland), QPM from Australia, C subtype 35 from Sweden, N10, N36, and N46 from Shanghai, China, LZ268, LZY79, LZ508, and LZ101 from Beijing, China and A21_p1177_sR3307, C36_p1075_s3911, and C43_p1154_sR1124 from Wisconsin (USA) based on VP4/VP2 regions (A) and 5′ NCR regions (B) of human rhinovirus. The trees were constructed by 'Neighbor-Joining' method in the MEGA 4 program and HEV-D 68 strain was used as outgroup. The distances of trees were computed using the Maximum Composite Likehood method and were the units of the number of base substitutions per site. The intraspecies recombination strains of HRV-A, interspecies recombination strains of HRV-A, intraspecies recombination strains of HRV-C and interspecies recombination strains of HRV-C are labeled using white circles, black circles, white squares and black squares, respectively. (DOCX) [file pone.0068081.s001.docx]

**Figure S1A**

**Figure S1B**
