# Supplementary material for: Identification of Recombinant Human Rhinovirus A and C in Circulating Strains from Upper and Lower Respiratory Infections
Source: PLoS One. 2013 Jun 27;8(6):e68081. doi: 10.1371/journal.pone.0068081 (PMC3695095; doi:10.1371/journal.pone.0068081)
Supplement: Figure S2 — The splits trees were predicted by decomposition network method in SplitsTree 4 (http://www.splitstree.org). 19 recombination strains showed the interconnected relationship in the network and supporting recombination between them. (A) The intraspecies recombinant strains of HRV-A, (B) interspecies recombinant strains of HRV-A, (C) intraspecies recombinant strains of HRV-C, (D) interspecies recombinant strains of HRV-C. (DOCX) [file pone.0068081.s002.docx]

**Figure S2A**

**Figure S2B**

**Figure S2C**

**Figure S2D**
